# Tendon Tissue Repair in Prospective of Drug Delivery, Regenerative Medicines, and Innovative Bioscaffolds

**DOI:** 10.1155/2021/1488829

**Published:** 2021-11-16

**Authors:** Muhammad Nadeem Hafeez, Nicola d'Avanzo, Valentina Russo, Luisa Di Marzio, Felisa Cilurzo, Donatella Paolino, Massimo Fresta, Barbara Barboni, Hélder A. Santos, Christian Celia

**Affiliations:** ^1^Department of Pharmacy, University of Chieti-Pescara “G. d'Annunzio”, Chieti 66100, Italy; ^2^Drug Research Program, Division of Pharmaceutical Chemistry and Technology, Faculty of Pharmacy, University of Helsinki, Helsinki 0001, Finland; ^3^Unit of Basic and Applied Biosciences, Faculty of Bioscience and Agro-Food and Environmental Technology, University of Teramo, Teramo 64100, Italy; ^4^Department of Health Science, University of Catanzaro “Magna Græcia”, Catanzaro 88100, Italy; ^5^Department of Clinical and Experimental Medicine, University of Catanzaro “Magna Græcia”, Catanzaro 88100, Italy; ^6^iDelivery, Nano&Bio Technologies, Reggio Calabria 89100, Italy; ^7^Helsinki Institute of Life Science (HiLIFE), University of Helsinki, Helsinki 00014, Finland; ^8^Department of Biomedical Engineering, University Medical Center Groningen/University of Groningen, W.J. Kolff Institute for Biomedical Engineering and Materials Science Ant. Deusinglaan 1, 9713 AV Groningen, Netherlands

## Abstract

The natural healing capacity of the tendon tissue is limited due to the hypovascular and cellular nature of this tissue. So far, several conventional approaches have been tested for tendon repair to accelerate the healing process, but all these approaches have their own advantages and limitations. Regenerative medicine and tissue engineering are interdisciplinary fields that aspire to develop novel medical devices, innovative bioscaffold, and nanomedicine, by combining different cell sources, biodegradable materials, immune modulators, and nanoparticles for tendon tissue repair. Different studies supported the idea that bioscaffolds can provide an alternative for tendon augmentation with an enormous therapeutic potentiality. However, available data are lacking to allow definitive conclusion on the use of bioscaffolds for tendon regeneration and repairing. In this review, we provide an overview of the current basic understanding and material science in the field of bioscaffolds, nanomedicine, and tissue engineering for tendon repair.

## 1. Introduction

Tendon is a viscoelastic connective tissue interposed between bones and muscles with the primary function to transmit the force generated during striated muscle contraction to the skeleton, thus allowing the joint movement. Based on this anatomical structure, tendon tissue is strongly stressed throughout the lifespan and must sustain extreme stress up to 100 MPa (megaPascals). Unfortunately, despite tendon capability to withstand huge tensile strength, these continuative solicitations can lead to several injuries (such as microtrauma or rupture) which, due to the hypovascularity and hypocellularity of tendon tissue, show exceptionally slow natural healing processes [1]. The accurate tendinopathy incidence worldwide is hard to evaluate, but it is estimated that around 30% musculoskeletal pain situations are related to tendon injuries. In particular, the highest incidence of tendinopathy was found in the older population and people involved with extreme musculoskeletal mechanical stresses, like athletes. Indeed, the latest estimations demonstrated that tendinopathy is involved in more than 50% of sport injuries [1, 2].

Currently, several conventional approaches are available for tendon repair and, especially in acute tendon injuries, they often require tissue grafts to accelerate the healing process. Unfortunately, in case of allografts, the problem of histocompatibility and tissue rejection has to be faced, although this shortcoming has been overcome with allograft autologous sources. Unfortunately, despite this last approach significantly decreasing the immune rejections, it is still a challenge to design fully compatible and functional autograft for injured tendons [3, 4].

In this scenario, regenerative medicine may play a crucial role by aiding to obtain functional grafts for efficient and faster healing of the injured tissues [5]. Regenerative medicine and tissue engineering are emerging interdisciplinary technologies that by combining cells, degradable polymers, and immune modulators can help to develop functional derivatives for several tissues, the periodontium [6], heart [7], tendon [8, 9], etc. In particular, scaffolds are an effective technological option for chronic and acute tendon repair, allowing at the same time an improved healing rate and high quality/functionality of repaired tissue. In these attempts, scaffolds with suitable mechanical biofunctional properties can be surgically implanted at the injured site in order to recapitulate the events for tendon tissue regeneration [10]. Ideally, this physical support should not only improve the cell attachment but also enhance the interactions between seeded cells and biomaterials thus controlling further cellular activities like cell proliferation, migration, and differentiation [11, 12]. In order to provide these functions and avoid side effects, several characteristics are required for 3D scaffold: nontoxic degradation products, biocompatibility, compatible degradation rate with host tissue growth, porosity, and mechanical strength [13, 14]. Several natural and synthetic polymers are currently in use to fabricate 3D scaffolds with enormous processing flexibility. Natural polymers like gelatin, chitosan, alginate, collage, and synthetic polymers, such as poly(lactic-*co*-glycolic acid), polylactide, polycaprolactone, polyurethane, and poly(glycerol sebacate), are common biomaterials used for tissue engineering [15]. Based on the wide range of materials available to date, the selective process plays a pivotal role, and it is influenced by several parameters like biodegradability, compatibility, severity of injury, and type of tendon tissue [16–18].

However, despite these promising characteristics, there are several questions to be investigated yet and the development of a safe bioactive scaffold shows suitable mechanical/biophysical properties, able to provide at the same time an adequate physical support for cell proliferation and differentiation and support the regeneration and cure of injury tissue. For example, in case of flexor tendon repair the use of appropriate scaffolds appears difficult, because of their size and minimum space left at the site of implantation with synovial sheath [19, 20]. However, rotator cuff and Achilles' tendon injuries have successfully been treated by using available bioscaffolds in combination with seeded tenocytes and growth factors [21].

In this review, we summarize the last advancements in tendon regeneration. We focused our attention on stem cell therapy and different materials used for scaffold construction highlighting the advantages of resulting 3D scaffolds as delivery systems for growth factors, cells, and/or genes compared to conventional therapies. Finally, we described the applications of nanocarriers in tissue engineering and the potential giant step forward which the combination of these two technologies (nanoparticles and scaffolds) may provide to tendon regeneration.

## 2. Structure and Function of Tendon

Tendon has a flexible structure that binds muscles to the skeleton and is composed of connective strong fibrous tissue able to resist tensile loading. As mentioned above, tendons provide a point of connection between the muscle and bone and transmit the force of muscle contraction allowing movement [1].

**Figure 1:**
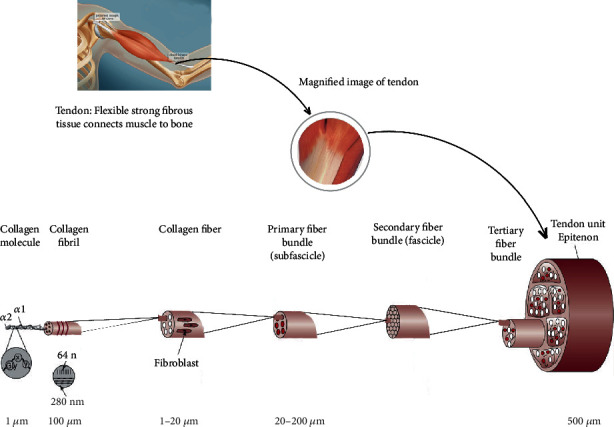
Hierarchical organization of type I, II, III, V, and XI, fibrillar collagens, and basic structural framework of tendon tissue. The triple helical structure of collagen is composed of 3-*α* chains each consisting of 1,000 amino acids rich in proline and glycine. Reproduced with permission from reference [22].

Tendons vary in size and shape. The histological structure of tendons exhibits wavy crimp and wavy sinusoidal pattern of collagen fibers in stretched and unstretched form, respectively. The structure of tendons demonstrates hierarchical organization of type I, II, III, V, and XI, fibrillar collagens (triple helical), and basic structural framework (Figure 1).

The collagen content of dry mass is 75-85% with type I collagen 95% and type III and V 5%, while the elastin content is about 3% of dry mass, glycoproteins, glycosaminoglycans, and proteoglycans constitute about 2% [23].

In particular, the two attachment sites (junctions), myotendinous and osteotendinous (also known as enthesis), present in every tendon are the most vulnerable tendon's site to injuries [24].

## 3. Tendon Injuries and Its Types

**Figure 2:**
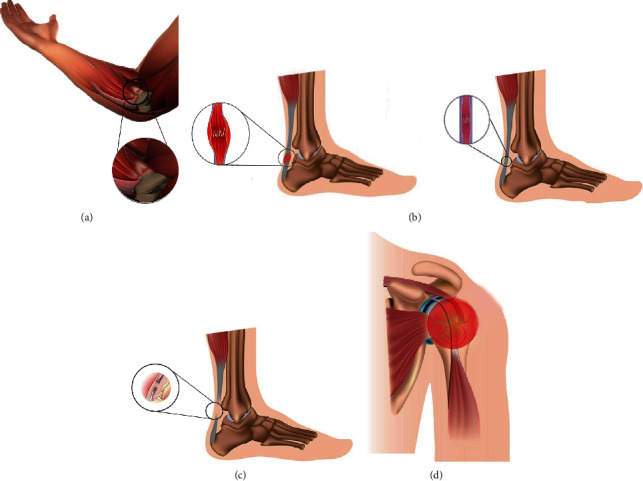
Major tendon injuries: (a) tendonitis, inflammation and irritation of tendon tissue, it is short term discomfort, usually caused by body's immune response; (b) tendinosis, long term issue and chronic pain without inflammation, condition share many symptoms with tendonitis but greatly different in case and appearance, it is common in the shoulder, elbow, hip, knee and Achilles' tendons; (c) tendon rupture, acute condition, complete break off tendon tissue mainly because of overuse or unusual overload and exercise, surgical intervention; (d) rotator cuff injury, tear off rotator cuff tendon that stabilize shoulder, acute condition. Reproduced with permission from references [31–33].

There are two major types of tendon injuries and acute processes. Acute injuries generally occur after sudden trauma, especially in people who are participating in active athletic activities, while chronic tendon injuries typically take place in aged patients after repetitive failure of mechanical events and persistent inflammation, and a late diagnosis may result in permanent disability [25, 26]. Sometimes acute tendon injuries are treated with nonoperative and conventional physiotherapy, such as closed injuries of wrist tendons while acute flexor tendon injury is predominantly treated by surgical intervention [26]. It is generally accepted that recurrent microtraumas occurring in fibrotic-healed tendons becomes a common condition that can lead to chronic solicitation up to ruptures [27]. Microtraumas are frequently associated with inflammation states, which have a core role in tendon pathology [28]. Inflammation has also historically influenced the tissue pathology classification, but currently the terms ‘tendinitis' and ‘tendinosis' have been recognized as an oversimplification, and tendinopathy is currently the best generic descriptive term for the clinical conditions in and around tendon disorders [29]. Moreover, the tendon healing process is also influenced from its anatomical position and functions, and the natural reparative processes of injured rotator cuff tendon was found often slow because of multidirectional joint motion, hypovascularization, and complex anatomical structure [30]. Major types of tendon injuries are shown in Figure 2.

One of the tendon tissues most involved in injuries is Achilles' tendon, in response to the extreme stresses to which it is subjected [34]. The major cause of Achilles' tendon injury is trauma, but chronic injuries are also frequently observed. Acute Achilles' tendon injury mainly takes place in highly active young individuals, usually sport men, when their tendon tissue is subjected to unusual trauma. Surgical treatment is recommended for patients who intend to continue with athletic activity after recovery, because the rerupture rate is found minimum with operative intervention as compared with conservatively treated injury [35, 36]. Microinjuries and failure of natural healing response are considered the foremost reasons behind Achilles' tendinopathies. The mechanism that stimulates microinjury is poorly understood although it is assumed that it fails to induce sufficient inflammatory response to accelerate standard triphasic natural healing process, thus leading to tendinopathies from moderate to severe ones, up to the complete rupture [30].

## 4. Natural Healing of Tendon Tissue

The triphasic natural healing response is comparatively slow, because of the hypovascular and hypocellular nature of tendon tissue, thus inviting surgical intervention [37]. The natural healing response is divided into three phases: (i) inflammatory, (ii) proliferation/repair, and (iii) remodeling [38]. During the inflammatory phase, blood clot formed immediately following injury acts as “preliminary scaffolds” and ruptured tendon vessels release chemoattractants for migrating cells (monocytes, neutrophils, and lymphocytes) from surrounding tissues [39]. During this step, digestion of necrotic debris carried out by phagocytosis and activation/recruitment of tenocytes is also initiated. After two days of injury, the second phase (proliferative phase) takes place. Fibroblasts migrate to the injured site and start to proliferate at epitenon and, simultaneously, intrinsic tenocytes from epitenon and endotenon reach at wound site and start proliferation. At this stage, the level of neutrophils is declined, and growth factors are continuously released by macrophages [39]. The ECM synthesis is being started by tenocytes, showing high contents of type III collagen, glycosaminoglycan, and water [40–42]. After 1-2 months of injury, the last remodeling phase starts. The amount of type I collagen is increased with consequently decrease in collagen type III, glycosaminoglycan, and cellularity of injured site. At 10 weeks, collagen fibers aligned in direction of stress/load, and it slowly changed into scar-like tendon tissue which never attains mechanical and structural properties like uninjured tissue even after 48 weeks of injury [43, 44].

Natural healing has three major issues: (i) cell infiltration sources, intrinsic (injured tissue) and extrinsic (surrounding tissues, e.g., synovial sheath). In particular, the extrinsic cellular infiltration contributes to the appearance of adhesion formation and scar-like tissue, which has abnormal structural and biomechanical properties and can results into gap at tendon-muscle junction (myotendinous) which affects greatly the strength and moth generated by muscle [45]. Indeed, resulting repaired tissue shows abnormal thickness, shape, and length that are all parameters which reduce its functionality [46]. Based on this, some reports maintained that operative intervention is better as compared with conservative treatment because it strongly reduced the nonfunctional scar formation [26], though some tendon tears were rehabilitated without operative repair like partial tendon injuries [46–48].

## 5. Conventional Treatment Strategies

**Figure 3:**
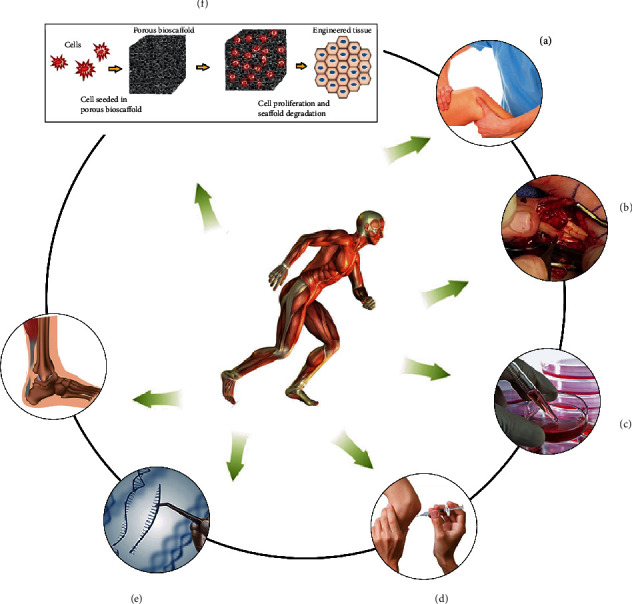
Different treatment options for tendon repair: (a) physiotherapy facilitates faster healing and collagen realignment, preventing joint stiffness, resulting in elevated tensile strength, and better gliding; (b) surgical intervention is usually used and has several disadvantages such as poor integration, mechanical mismatch, necrosis, donor site morbidity tissue rejection, and disease transmission; (c) stem cell therapy facilitates tendon tissue regeneration process; (d) growth factor therapy aims attract stem cells at injury site, support attachment, proliferation, differentiation, and accelerate tendon regeneration; (e) gene therapy aspires to synthesize natural proteins that overcome the problems of externally delivered growth factors; (f) regenerative medicines (tissue engineering) accelerate natural healing process and design bioscaffolds (natural, synthetic, and composite) for the rehabilitation of tendon tissue, to deliver growth factors, to stimulate cell proliferation and differentiation. Reproduced with permission from references [53–57].

Currently, chronic and acute tendon injuries are commonly treated either with conservative treatment approaches or surgical intervention. Conservative treatments such as corticosteroid injection, rest, orthotics, laser treatment, and ultrasound are frequently used, and they provide pain relief. On the other hand, surgical intervention may be required when satisfactory results were not attained with conservative approaches [49, 50]. Operative interventions are frequent in acute injuries, but the quality of repaired tendon remains inferior in terms of structure and functionalities compared to noninjured tissue, mainly because of misaligned collagen fibers and distorted composition of ECM. Besides scarring tissue and inherent risk of surgery, additional considerable risks like adhesion formation, infection, nerve damage, and risk of other diseases are associated with conventional treatment approaches. In these attempts, physiotherapy is often associated with surgery, providing a faster healing and proper collagen realignment [51]. In severe cases, biological grafts are used to replace damaged tissue. Autografts are frequently used approach to repair severely damaged tendons; however, it may cause functional disability and high morbidity at donor sites. Mechanical mismatch, poor integration, necrosis, and tissue laxity are considerable disadvantages of autograft therapy [52]. Allograft is an alternative to autograft, but this approach is also not free from risks like tissue rejection and disease transmission (Figure 3).

Several FDA-approved commercially prosthetic devices, since 1970, are available in the market as alternatives to autograft, but the continuous muscle contraction and mechanical load restrict prosthetic device applications as satisfactory substitutes [58]. In fact, results of these products, although satisfactory for the short term, are often associated with complications and ambiguous long-term results [59].

## 6. Materials Used for New Approaches to Tendon Repair

### 6.1. Materials for Tissue Engineering Approach

Tissue engineering is aimed at facilitating natural repair by the development of synthetic graft *in vitro* that can be implanted at severe injured sites [60]. It plays an important role through synthetic grafts to improve the rehabilitation strategies and tendon repair management [61]. In these attempts, scaffolds have been the most common strategy investigated for tissue repair to date [21]. Tissue engineering and scaffolds are aimed at preventing rerupture and minimizing inflammation by providing mechanical support to accelerate healing tendon process, by facilitating cell recruitment at wound site, promoting cell proliferation, and stimulating ECM production and the proper organization of collagen fibers [22]. Preliminary studies support the idea that scaffolds can provide an alternative to conventional treatments for tendon augmentation with an enormous therapeutic potentiality [10]. However, available data are lacking to allow definitive conclusion on the use of scaffolds for tendon augmentation. Cell attachment, proliferation, differentiation, ECM formation, diffusion of metabolites, and alignment of collagen fibers are foremost desirable properties of scaffolds in tendon tissue repair. The interaction between seeded cells and scaffolding materials is a key to success towards designing functional scaffolds. Ideally, scaffolding materials must stimulate regenerative processes providing basis for the proper ECM deposition, inducing at the same time a suitable cell differentiation and proliferation rate [62, 63].

Based on the materials used three major types of scaffolds are currently available to rescue the severe tendon injuries: (i) synthetic, (ii) biological, and (iii) composite scaffolds.

#### 6.1.1. Materials for Synthetic Scaffolds

Synthetic scaffolds are augmented grafts of synthetic material like polyglycolic acid (PGA), polylactic acid (PLA), carbon fibers, teflon, decaron, polybutyric acid, and bioactive glass. They have suitable mechanical properties and less immunogenic reaction but limited biocompatibility compared to scaffolds made up of natural materials [58, 64]. Indeed, synthetic scaffolds are typically more versatile in terms of physicochemical and structural properties compared with biological ones because they can be synthesized under specific conditions [65, 66]. Although synthetic scaffolds provide promising results, for instance, the lack of signaling molecules and mechanical brittleness restrict their wide range of applications in tissue engineering [67, 68]. Several polyesters such PLA, PGA, and PLGA have widely been explored for tendon tissue repair. Lactic acid and glycolic acid are products of their degradation, which are secondary metabolites of the body that further enhance their biocompatibility. Cooper et al. demonstrated that PLGA is a good choice as a scaffolding material for Achilles' tendon repair [64]. Moreover, recently, it has been demonstrated that electrospun highly aligned PLGA tendon biomimetic scaffolds, which resembled collagen fibers of the tendon ECM, were able to induce an early epithelial-mesenchymal transition (EMT) and tenogenic differentiation of amniotic epithelial stem cells (AECs). The use of this type of stem cells allowed to verify in depth the topological effect of the scaffolds and the mechanisms that permitted to an epithelial cell (having a cuboidal shape and generally not expressing collagen type I) to differentiate towards the mesenchymal tenogenic lineage [69, 70]. These findings suggested a beneficial implication of PLGA in tendon regeneration, exhibiting satisfactory collagen production and proper mechanical properties, enhanced histological scores, and facilitated speedier wound healing [71]. PGA was also reported as a feasible scaffolding material to restore mechanical strength of repaired tendon tissue in a hen model [19]. Indeed, degradation time has been increased in case of woven scaffolds of PGA surpassing mechanical performance as compared with unwoven PGA scaffolds [50, 72]. Despite sharing the single group of polyhydroxyesters, PLA, PLGA, and PGA were found quite different in their degradation profile and their cellular responses at molecular level. This difference has been reported by Liu et al. by comparing three different scaffolding materials: PLGA, PGA, and poly L-lactic acid (PLLA) [37, 73].

**Figure 4:**
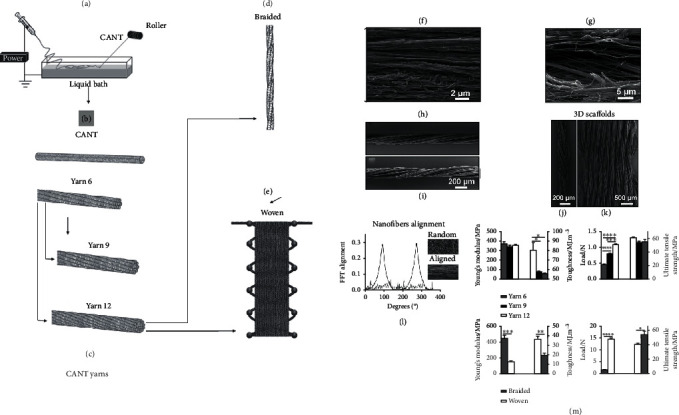
Hierarchical assembly of CANT: (a) electrospinning system, (b) elementary unit of 3D assembly, (c) yarn composed of twisted CANT, (d) braided 3D scaffold, (e) woven 3D scaffold, (f, g) SEM images of CANT observed at 0.02 ms^−1^, (h, i) SEM images of yarn, (j) braided 3D scaffold, (k) woven 3D scaffold, (l) degree of nanofiber alignment in CANT and random mess, (m) tensile properties of yarn 6, 9, 12, and 12, and (o, p) tensile properties of braided and woven 3D scaffolds. Reproduced with permission from reference [74].

Another synthetic material used in tendon tissue engineering is poly-*ε*-caprolactone (PCL). In these attempts, 3D hierarchical scaffold seeded with human adipose stem cells (hASCs) and human tendon-derived cells (hTDCs) has been fabricated by electrospun nanothreads (CANT) composed by purified chitosan and PCL. The use of these scaffolds made up of aligned fibers led to a tendon-like nano-to-macro architecture and high expression of tendon-related (Figure 4) markers (Col type I and type III, Ten-C, and Scx) for both investigated cell types as compared with the control [74].

**Figure 5:**
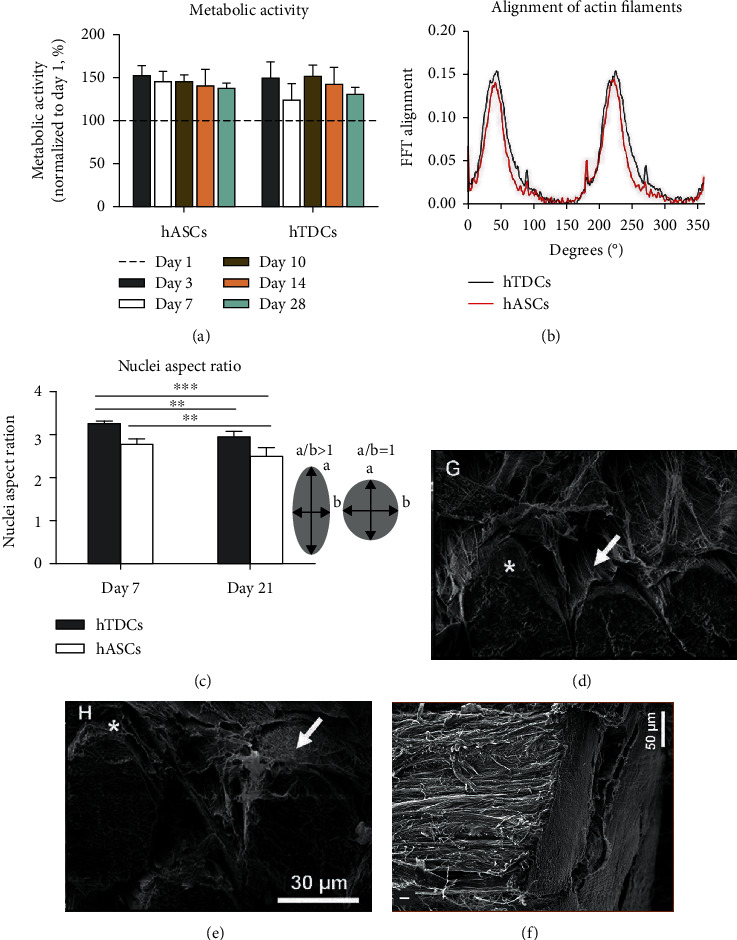
(a) Metabolic activity of seeded human adipose stem cells (hASCs) and human tendon-derived cells (hTDCs) between 1 and 28 days, (b) frequency plot of actin filament alignment at 21 days between hASCs and hTDCs, (c) nuclei aspect ratio of hASCs and hTDCs, (d) SEM image 3D scaffold seeded with hTDCs showed complete covering of ECM, (e) SEM image 3D scaffold seeded with hASCs showed covering of ECM, and (f) SEM image of bovine posterior ligament. Reproduced with permission from reference [74].

In the same study, 3D hierarchical scaffold seeded with hASCs (PCL and chitosan reinforced with hydroxyapatite and glutaraldehyde) was constructed by wet spinning technique (Figure 5), demonstrating to mimic the topographical and mechanical properties of the native tendon-to-bone interface [74].

Regardless of several advantages of polyesters, there are still numerous limitations awaiting to be addressed. For example, PGA scaffolds lost their mechanical strength because of their bulk degradation profile which resulted in loss of integrity and matrix disruption [75]. Furthermore, the two main limitations in the use of polyesters as a raw material for scaffold preparation are related to their hydrophobicity and degradation products. Indeed, their hydrophobic nature does not support required adhesion of stem cells and therapeutic nanoparticles, that is, a crucial step during the engineering of compatible and functional scaffolds [76, 77]. Moreover, despite the byproducts (degradation products) of polyesters being usually natural metabolites and acidic in nature, the presence of these products in high concentration may cause disturbance in normal metabolism and homeostasis at implantation site [69]. The first limitation may be overcome by surface modification of polyester scaffold with fibronectin [78, 79] as a strong adhesive agent. Second limitation still forces the application of polyester scaffolds for repairing of smaller tendon injuries because with smaller scaffolds; the adverse effects of secondary metabolites are generally reduced.

#### 6.1.2. Materials for Biological and Composite Scaffolds

Biological scaffolds are derived from bovine, porcine, equine, and human tissues by decellularizing the extracellular matrices [80, 81]. They also took origin from different biological materials like collagen, fibrin, gelatin, hyaluronan, agarose, alginate, and chitosan [80]. Bio-BlanketW®, derived from bovine dermis, OrthADAPT®, derived from equine pericardium, and Restore®, synthesized from porcine small intestine mucosa, are FDA-approved scaffolds, currently available on market for tendon tissue repair [58]. In these scaffolds, the dermis, pericardium, and intestine mucosa, respectively, are processed by removing noncollagen and cellular components [58, 82]. Scaffolds, derived from the small intestinal submucosa, have successfully been used for treatment of Achilles' tendon and rotator cuff injuries [83]. Allografts can be also *in vitro* recellularized providing proper scaffold for tendon tissue repair [84, 85]. These scaffolds have several advantages like natural structure, biomechanical stability, and strength over synthetic allografts [84]. Indeed, ECM of decellularized allografts is considered nearer to natural tissue in terms of cell proliferation, cell attachment, mechanical stimulation, and diffusion of metabolites [83, 86].

Given that collagen is the main component of ECM, biological scaffolds derived from collagen are highly compatible, considered superior choice as compared with polyesters-based synthetic scaffolds. These scaffolds have extensively been investigated for tendon tissue repair applications, demonstrating a better cell adhesion capacity and cell proliferation ability compared to synthetic ones. In these attempts, improved quality of repaired patellar tendon injury has been reported with collagen gel [86]. However, collagen-based scaffolds show inferior mechanical strength compared to synthetic polyesters. In order to overcome this limitation, collagen gel has been combined with polyglyconate suture, thus showing improved biomechanical properties of repaired patellar tendon in the hen model as compared with the control, although it was far inferior to uninjured tendon [87]. Moreover, physical support, obtained by combining aligned collagen fibers with collagen gel or sponge, demonstrated a higher cell seeding capacity compared with a random collagen gel [88]. Collagen sponges and fibers also exhibit superior mechanical strength, compared with the collagen gel and the combination of these as a scaffolding material provide further appealing substitute of allografts and polyesters-based scaffolds [89]. Apart from the poor mechanical strength of collagen [88] being overcome by combining it with other materials [58, 90], other restrictions in the use of this polymer are related to its difficult characterization due to the several limitations in its processability and to the possibility to induce immunogenic reactions [91].

Agarose, alginate, chitosan, and chitin are also widely studied for tissue engineering, despite them having traditionally been considered for hard tissue regeneration as a scaffolding material. They remained underutilized for soft tissue engineering; however, recently, they got significant attraction as a possible scaffolding material for the cartilage and tendon tissue repair [92, 93]. Particularly, chitosan got tremendous significance to be used as a scaffolding material in the field of soft tissue engineering especially tendon regeneration, exhibiting hydrophilic nature, superior mechanical strength, better cell attachment, and proliferation properties as compared with hydrophobic polyesters PGA and PLA [94]. Chitosan is a linear polysaccharide, deacetylated product of chitin, composed of N-acetyl-D-glucosamine and *β*-1–4-D-glucosamine randomly distributed units. Own enhanced cell attachment, proliferation, differentiation, highly porous structure, and ECM production make chitosan a suitable candidate for a scaffolding material in the tendon injury. In particular, chitosan was found to exhibit superior biofunctionality because of the presence of N-acetylglucosamine moiety, an analogue of glycosaminoglycan, which provides enhanced adhesion capacity to growth factors and other proteins [94]. Porous chitosan scaffolds were designed with microchannels to engineer patellar tendon tissue, exhibiting optimal results in terms of histological and biomechanical scores [92]. Combination of chitosan with other polysaccharides has also been explored: the combination of chitosan with hyaluronan (HA), an essential component of ECM, enhanced mechanical capability, and cell migration, adhesion, and differentiation [93]. The hyaluronan-chitosan scaffold also improved the production of collagen type I in the rotator cuff regenerated tendon [35, 93, 95].

Another polysaccharide, alginate, can be used in combination with chitosan as a scaffolding material because it contains D-glucuronic acid that is considered an analogue of glycosaminoglycans having similar biological activities. Chitosan-alginate hybrid scaffold showed significantly enhanced cell adhesion to tenocytes and production of ECM, predominantly made up of collagen type I [96]. Similarly, combination of nanohydroxyapatite (n-HA) particles with fibrin, chitin, gelatin, PCL, PLGA, PLA, and polyamide-based composite scaffolds has been explored for tendon repair [97, 98]. These studies highlighted that the combination of biological and synthetic biomaterials for hybrid scaffolds is a promising approach towards tendon repair technology [58, 90].

### 6.2. Limitations of Scaffolds in Tendon Tissue Repair

Although scaffolds provide promising results for tendon tissue engineering, they still have some limitations, which restricts their applications. The major problem with scaffolds is cell source and their *ex vivo* regeneration [99]. After seeding cells on the scaffold, they are regenerated by two methods: (i) in bioreactor, *ex vivo* reconstruction and (ii) by implantation in the body, *in vivo* reconstruction. In recent decades, efforts have been paid on *ex vivo* regeneration of the cells to expand tissue engineering business. Indeed, mass production of engineered tissues could offer products that can be delivered to medical centers on their demand; however, the cell source is not from patients but from healthy active individuals, thus highlighting several concerns in terms of safe clinical applications of these devices. Furthermore, up till now, no standard harvesting, seeding, and maintaining protocols are designed yet, and seeded cells behaved differently *in vitro* and *in vivo* [100, 101]. In order to prevent the risks of contamination and disease transition, the standardization for the safety assessment of the cell seeded scaffold constructs is required, although difficult because human cells have never been a therapeutic object of sales. Moreover, several studies reported restricted diffusion of essential metabolites and products included into scaffolds *in vitro*, pointing the attention on another key problem that needs to be solved: the neovascularization processes *in vivo* [64, 102].

## 7. Gene Delivery Systems

Gene therapy can be used for tendon repair to aid synthesizing proteins that overcome the problem of immune response and short life span of growth factors [103, 104]. However, this approach also has disadvantages, such as high degradation rate of RNAi/DNAi by immunocytes, as well as risks associated with adenovirus (that is used for gene transfection) are extensively debated [105]. Several studies have been carried out focusing on gene delivery methods and materials for healing and regeneration of tendon tissue (Table 1).

Full-length transformation of genes, carried by fibrin gel, encoding fibromodulin to rat Achilles' tendon laceration model, demonstrated an enhanced healing response with better biomechanical properties of repaired tissue [106]. Similarly, adenovirus promoted the transfection, and then, the expression of BMP-14 into the rat Achilles' tendon laceration model, which exhibited 70% greater tensile strength and increased cellular proliferation of tenocytes at 2 weeks postrepair as compared with control [107].

Considering associated risks of transfection, other delivery materials have also been investigated instead of adenovirus, such PLGA nanospheres that can incorporate plasmids and effectively deliver RNAi/DNAi into tenocytes [72, 108]. The use of smart biomaterials acting as interfaces to enhance the temporal and spatial presentation of genes in the target place and/or acting as scaffolding material is an innovative approach to overcome shortcomings that restrain the efficacy of growth factors and stem cells [109]. Polymersomes and liposomes that are biocompatible and safety nanocarriers having different therapeutic applications [97] were also used as delivery systems for fibromodulin encoding gene to rat Achilles' tendon laceration model and demonstrated improved mechanical tendon strength [106]. This approach has also been explored to switch off (gene silencing) the expression of adverse proteins to control scarring during the healing process. For example, silencing the expression of decorin by shRNA transfection into rat patellar tendon cells demonstrated speedy and scar less healing of patellar tendon [110].

Although gene-based therapy has shown great potential of treating tendon injury and degenerative conditions, there are still concerns regarding the safety profile of genetic materials, such as potential mutagenicity immune reaction associated with the use of plasmids [111, 112] and viral vectors. Conversely, the nonviral vectors have a safer profile, but they have decreased transfection efficiency [113]. Further work needs to be carried out to advance the current field toward developing more effective transfection materials with either no or minimal toxicity. Current advances in gene therapy for tendon tissue repair are described in Table 1.

## 8. Growth Factor Delivery Systems

The delivery of growth factors/immune modulators is an emerging approach in regenerative medicine, in order to support the collagen synthesis and ECM synthesis and facilitate cell proliferation for tendon regeneration [114–116]. Several growth factors and their delivery strategy are currently being studied for this purpose such as insulin-like growth factor (IGF), transforming growth factor beta (TGF-*β*), vascular endothelial growth factor (VEGF), platelet-derived growth factor (PDGF), bone morphogenetic proteins (BMP), and basic fibroblast growth factor (bFGF) [117, 118]. Injection of TGF-*β* into native murine knee increased proteoglycan contents. The TGF-*β* signaling pathway includes SMAD2/3 and MAPK pathways which upregulate various factors that help in maintaining a tenogenic environment for regeneration [119, 120]. Although multiple advantages have been reported for local application of injectable growth factors at injured tendon tissue, there are still limitations. The major problems occurred are their short half-lives, the escape from application sites, and the degradation by the immune system which makes nanomedicine ineffective for long duration [121]. The restrictions of injectable growth factors need to develop an efficient delivery system that retains these factors in the targeting site and allowed a continuous and controlled release of payloads [1, 122].

To overcome these drawbacks, new bioactive scaffolds seeded with growth factors and stem cells were developed to enable cell migration and proliferation favorable for tendon regeneration *in situ*. For example, fibrin gel endowed with TGF-*β*3 was found to increase vascularity and cell proliferation and accelerate healing process, when implanted at injured supraspinatus tendon of rats, thus increasing the structural, biomechanical, and functional properties of resulting reparative tissue [120, 122]. A further investigation demonstrated that the porous network of knitted PLGA-fibrin gel embedding exogenous basic fibroblast growth factor (bFGF) and sheets of mesenchymal stem cells (MSCs) were shown to have the highest expression of tendon-related gene markers. Outstanding repair efficacy, including appreciable biomechanical strength and native-like histological microstructures, showed that the MSC sheets contributed directly to tendon regeneration and exerted an environment-modifying effect on the injuries *in situ*, consistent with the beneficial effect of bFGF. No immunological incompatibility and rejection were found on implantation in Achilles' tendon defect model [14, 117]. Another application of scaffolds as a growth factor delivery system was investigated by using biphasic silk fibroin scaffolds with heparin. The resulting therapeutic physical supports were found to increase the attachment capability of TGF-*β*2 and growth/differentiation factor 5 (GDF-5) to the scaffold matrix resulting in biological effects at lower doses. Combined impact of growth factors and pore alignment of silk fibroin scaffold on adipose-derived mesenchymal stem cells (AdMSCs) was also analyzed. TGF-*β*2 and pore anisotropy synergistically increased the expression of ligament/tendon markers and collagen I protein contents. In addition, combined delivery of GDF-5 and TGF-*β*2 enhanced the expression of collagen II protein and cartilage markers on substrates with isotropic porosity [123]. Similarly, dual growth factors, bone morphogenetic protein-2 (BMP-2), and platelet-derived growth factor-BB (PDGF-BB) immobilized on polycaprolactone (PCL)/Pluronic F127 porous membrane were found to significantly accelerate the regeneration of bone tendon interface (BTI) injury. Probably, this effect was due to the physical barrier caused by porous membrane and continuous release of both growth factors, thus leading to a complementary effect able to create a multiphasic structure (fibrocartilage, tendon, and bone) similar to 3D native structure [123, 124]. Implantation of collagen sponge coseeded with three growth factors bFGF, TGF-*β*1, and BMP-12 was also investigated in rat Achilles' tendon model exhibiting a rapid increase in mechanical strength and fast tendon remodeling [125].

Apart from the high cost of growth factors that strongly limit their clinical use, to date a large range of controversies exist regarding the number of injectable/seeded growth factors onto scaffolds. Generally, considering the complex mechanism needed to fully stimulate the healing process, it is likely to be recommended, different combinations of growth factors must be applied at one time, thus resulting in a further increase of therapeutic costs [124, 126].

Current advances in the immune modulator therapy for tendon tissue repair are described in Table 2.

## 9. Stem Cell Therapy

Stem cell therapy is an attractive approach in tendon regenerative medicine [125]. Cells obtained from different tissues have been widely used, including tissue specific cells, such as tenocytes [127, 128] derived from tendon or dermal tissues [37, 129] and either nonspecific tissue like mesenchymal cells [37, 130] derived from the adipose or bone marrow which were used as biomaterials for tendon regenerative medicine. Local injections at wound site showed promising results in clinical trials and 86% improvement in terms of reduction in pain was found, when autologous tenocytes were injected at wound site in 20 patients with chronic severe tendinopathies of extensor tendon [127]. Similarly, 91% improvement was reported in case of arms and shoulder disabilities through tenocytes injection. Unfortunately, tendon-derived tenocytes show several drawbacks mainly due to their limited availability and cause donor site morbidity. Therefore, dermal fibroblasts are considered an abundant and readily accessible cell source to address this limitation [131].

The ultrasound-guided injections of autologous dermal fibroblasts demonstrated to cause reduction in pain and severity of tendinopathy and improved functional scores in 46 patients suffering from refractory patellar tendinopathy [131, 132]. In another trial, 20 patients, suffering from refractory lateral epicondylitis, were successfully treated with injectable dermal fibroblast and exhibited tendon thickness and presence of tendon tissue resembling the native state. Highly organized and aligned collagen fibers are reported also in collagenase-induced tendinopathy in rabbit Achilles' tendon, when three doses of autologous tenocyte were injected at wound site [128].

Another promising cell source, i.e., mesenchymal stem cell (MSC) for tendon tissue repair, having multilineage differentiation and self-renewal ability derived from a variety of tissues: the adipose, tendon, and bone marrow. Indeed, the implantation of bone marrow-derived mesenchymal stem cell (BM-MSC) was both safe and effective for the treatment of tendinopathy. In these attempts, MSC were intralesional injected in 113 racehorses showing digital flexor tendinopathy. After 3 years, 98.2% had returned to racing and the reinjury rate was found lower when compared with conservative treatment approaches [133]. A similar trend was found in other equine models, where BM-MSC treatment has resulted in reduction of the reinjury rate and quicker recovery time [133–135]. Improved tendon stiffness and well-organized crimp structures were found when damaged digital flexor tendons of 12 racehorses were treated with autologous BM-MSCs. Improved histological scores were recorded, and these were accompanied by decreased water content, swelling, and MMP-13 activity [134].

**Figure 6:**
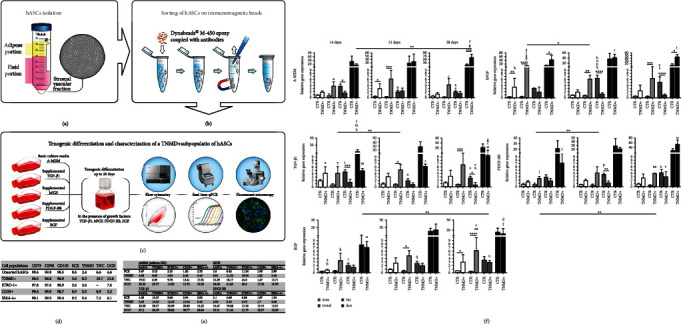
Schematic representation of steps to sort subpopulations of hASCs: (a) isolation of hASCs, (b) immunomagnetic sorting of hASCs, (c) tenogenic differentiation of TNMD + hASCs, (d) expression of tendon-related markers in sorted and unsorted subpopulations of hASCs, (e) response of sorted and unsorted subpopulations of hASCs to different growth factors, and (f) gene expression level of Tnmd, Scx, TNC, and DCN in sorted subpopulations of hASCs. Reproduced with permission from reference [136].

Gonçalves et al. also demonstrated that immunomagnetically separated subpopulation (TNMD^+^ cells) of human adult multipotent adipose-derived stem cells (hASCs) obtained from the stromal vascular fraction (SVF) of adipose tissue had more tenogenic differentiation capacity and Tnmd, Scx, TNC, and DCN gene expressions (Figure 6) as compared with control (unsorted hASCs) and other sorted cell (STRO-1+, CD29+, and SSEA-4+) hASC subpopulations (Figure 6).

**Figure 7:**
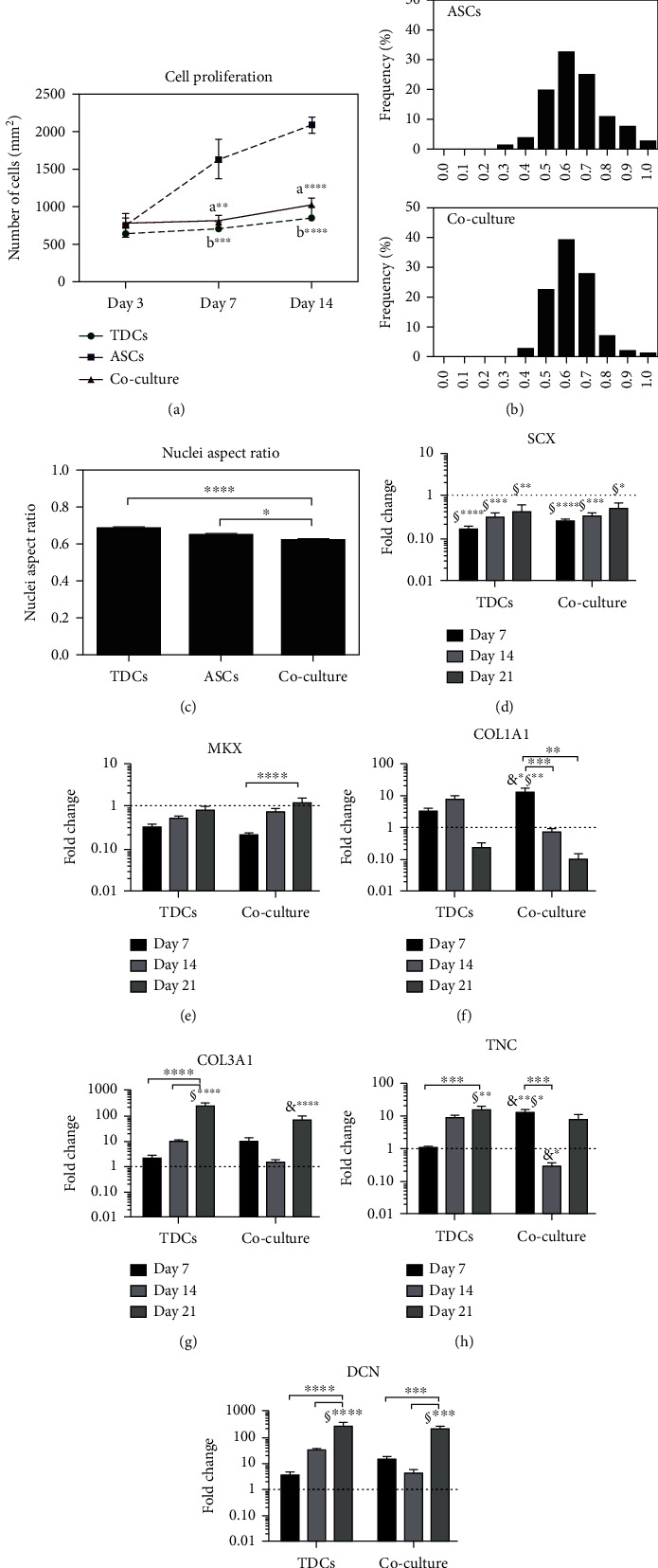
(a–c) Nuclear aspect ratio and cell proliferation at 3, 7, and 14 days; results are presented as mean ± SEM. (d–i) Expression of tendon-related genes in single and coculture conditions: (d) Scx, (e) MKX, (f) COL1A1, (g) COL3A1, (h) TNC, and (i) DCN. Reproduced with permission from reference [138].

Overall, TNMD^+^ subpopulation showed the highest number of positive cells for tendon markers at 28 days. This study concluded that the expression of tenogenic genes in TNMD^+^ subpopulation is increased as compared to unsorted hASCs with exception to the TNC gene where the trend was found opposite [136, 137]. TNMD^+^ cells showed an increased expression of Scx after 14 days in bFGF-supplemented media, while Tnmd was predominantly expressed at 21 days in basic and supplemented media with bFGF and TGF-*β*1; the DCN expression level was also increased in basal and bFGF-supplemented media (Figure 6). Furthermore, magnetic cell sheets, seeded with TNMD^+^ cells of human adipose stem cells (hASCs) coloaded with iron oxide nanoparticles (MNPs), demonstrated cell stability and improved mechanical and morphological properties and high expression level of tendon-related markers. In another study, establishment of direct interaction between human adipose-derived stem cells (hASCs) and native human tendon-derived cells (hTDCs) in the coculture system demonstrated the influence of hASCs on hTDCs in terms of controlled spontaneous cell elongation, proliferation, and high expression of ECM-related genes particularly Tnc, Col type I, MMP-1, MMP-2, MMP-3, and TIMP-1. Cells in coculture exhibited lower proliferation rate and were more elongated, and the highest proliferation rate was found for hASCs alone at 7 days (Figure 7). Nuclei of cells in coculture appeared to be more elongated, exhibiting significantly lower aspect ratio values in comparison to hASCs or hTDCs monoculture. Similarly, direct interaction between hTDCs and hASCs resulted in the enhanced expression of COL1A1, COL3A1, and TNC (Figure 7). Increased COL1A1 transcript levels were found in coculture conditions at 7 days as compared to hASCs and hASCs alone. This study concluded that hASCs may be good candidates in modulating the behavior of native tendon cells, particularly through a balanced process of ECM deposition [138].

Cell type widely studied and used for tendon regeneration in the last few years is AECs. This type of cells acquired an increasing role in tendon cell-based therapies thanks to the preclinical studies conducted either through allotransplantation or xenotransplantation approaches on injured calcaneal tendon in ovine model, and to the clinical trials carried out on spontaneous tendon lesions in horses [108, 137]. In fact, some studies have demonstrated that AECs can support tendon regeneration and an early recovery of the biomechanical properties of the tissue. Through these investigations, it has been elucidated that transplanted AEC support tendon regeneration partly through a paracrine stimulation of the damaged host tissue by modulating the production of critical growth factors (i.e., vascular endothelial growth factor VEGF and transforming growth factor beta 1 TGF-*β*1) and immune modulatory cytokines involved in healing processes. Interestingly, the obtained data, under allotransplantation and xenotransplantation settings, confirmed a direct role of AECs in the process of tendon regeneration through them *in situ trans*-differentiation towards the tenogenic lineage. This stem cell source is indeed able to direct tendon healing by stimulating a prompt recovery of tissue function without any preliminary transfection [139, 140]. Indeed, fetal tendon explants cocultured with AECs developed *in vitro* tendon-like three-dimensional structures in 28 days with a high expression profile of matrix (COL1 and THSB4) and tendon-related genes (TNMD and SCXB). Moreover, the produced tendon-like organoids displayed high levels of organization as documented by the cell morphology, the newly deposited matrix enriched in COL1, and widespread expression of gap junction proteins (Connexin 32 and 43) [140].

The interaction between hTDCs and pre-OBs (preosteoblasts) for enthesis regeneration was also explored in the coculture system. Higher transcription levels of bone- (ALPL, RUNX2, and SPP1) and interface-related genes (ACAN, COMP) and higher matrix mineralization were found in the coculture osteogenic medium [76, 77]. Current advances in stem cell therapy for tendon tissue repair are described in Table 3. Stem cell therapy safe for tendon regeneration still needs long-term studies. The major drawbacks of injecting stem cells at injured site are usually includes temporary swelling, pain, immune reaction, inability of cells to retain at injured site, and high morbidity which results in the low proliferation rate. Stem cell injections carry the same risks as any other therapeutic injection, such as a small risk of infection [141, 142]. To avoid immune reaction, generally, autologous cells are preferred. The patient is at a higher risk of an unwanted reaction if the stem cells are harvested from another person or animal and cultured in lab. Sometimes, injection of immune modulators and other therapeutic drugs along with stem cells to enhance their proliferation rate may also cause another risk factor. Some research advocates that foreign, manufactured, and engineered stem cells may elevate the risk of tumors. For this reason, the FDA limits how much stem cells can be manipulated and their minimum dosage at injured site [142, 143].

## 10. Mechanical Stimulation

**Figure 8:**
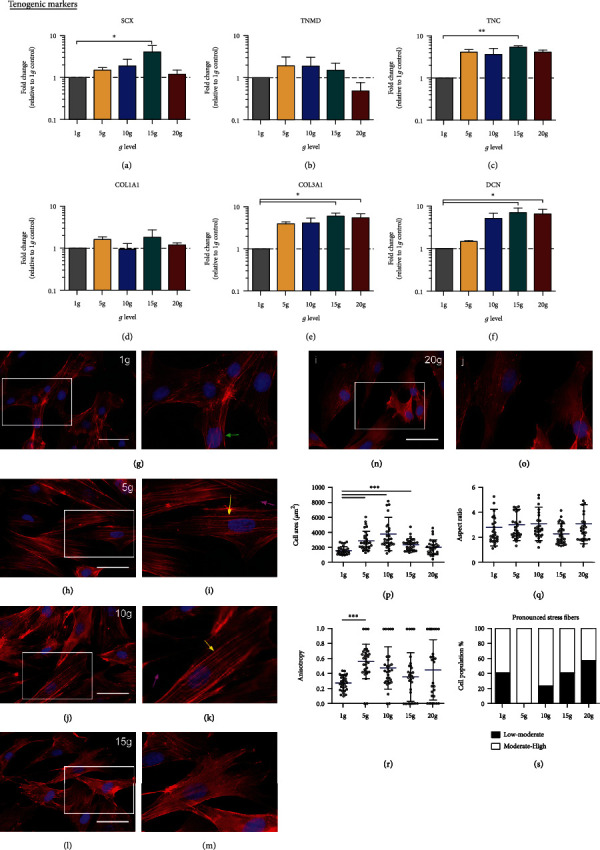
Transcription level of tendon-related genes: (a) SCX, (b) TNMD, (c) TNC, (d) COL1A1, (e) COL3A1, and (f) DCN. Effect of hypergravity at 16 h on morphology and F-actin distribution: (g–o) organization of F-action under normal and hyper-gravity conditions, (p, q) quantification of cell surface area, (r) measurement of anisotropy, and (s) quantification of stress fibers in hTSCs. Reproduced with permission from reference [154].

Molecular and cellular basis of drug-controlled loading was extensively explored and found improved mechanical properties of healing tendon, although optimal magnitude and time is still under debate [144, 145]. Inferior mechanical properties were found in healing tendon of animal models with zero mechanical stimulation, while overloading is also harmful to natural healing response [144, 146]. Given the ambiguous evidence in favor of optimal protocol for mechanical stimulation, it is generally accepted that healing tendons must be loaded in a controlled manner for expected results [144]. Increased proliferation, gene expression, and collagen production are found in exposed mesenchymal stem cells (MSC) and fibroblasts to uniaxial tension [147]. Unregulated Scx-gene expression was discovered by Scott et al. in MSC (cell line C3H10T1/2) exposed to cyclic strain [148]. It is anticipated that mechanical stress induces tenogenesis via the TGF-*β*-dependent biochemical and integrin-dependent signaling pathway [149, 150]. Chronic tendon injuries are the outcome of overexpression of inflammatory and catabolic mediators [50, 151]. Inflammatory products like MMP-1 and COX-2 expressions and production of PGE2 are found to be decreased when fibroblasts were subjected to uniaxial stretching up to 4% while increase after higher stretching (8%) [150, 152]. Similarly, tenogenic differentiation was found to be increased in TDSCs at 4% cyclic stretching; however, the increase in stretching causes enhanced production of osteogenic, chondrogenic, and adipogenic differentiation [147, 153]. In view of this discussion, it is recommended, controlled loading on healing tendons that promote tenogenesis, ECM production, and alignment of collagen fibers in the direction of loading, resulting in better mechanical and biochemical properties of healed tendon tissue. Gravity influences biological and physical processes, thus having an impact on homeostasis of living systems. The musculoskeletal system is composed of several mechanically responsive tissues and altered gravity influences the properties of skeletal muscle development. Exposure of musculoskeletal tissues to hypergravity may constitute a way of simulating (over)loading or, eventually, to be used as a measure to rescue cell phenotype after exposure to near-weightlessness conditions [103]. Effects of hypergravity (5, 10, 15, and 20 g) on the viability of hTDCs and expression of tendon-related genes were evaluated. It was found that the expression of scleraxis (Scx), tenascin (TNC), decorin (DCN), and III (COL3A1) was significantly increased by 4-, 5.4-, 6.4-, and 7-folds, respectively, at 15 g after 16 h. However, no difference was observed in the transcription level of tenomodulin (TNMD) and collagen type I (COL1A1) as compared to the control (Figure 8). It was also demonstrated that hypergravity (5, 10, and 15 g for 16 h) has an influence on morphology, anisotropy, and organization of actin cytoskeleton of cells (Figure 8). These findings opened new perspectives for research focused on using altered gravitational force as a model for (over)loading as a tendon tissue engineering approach [154].

## 11. Nanoparticles and Tendon Regeneration

From the last decade, there was growing interest to synthesize nanoparticles for tendon regeneration and treatment. Nanomaterials are proposed to be a potential breakthrough in tendon regeneration technology in terms of improvement towards drug delivery (growth factors), gene therapy (as gene carrier), cell proliferation, anti-inflammatory, antiadhesion, antimicrobial properties, and enhanced physicochemical and morphology of repaired tissue [155, 156].

The size of nanoparticles is usually in range of 20 and 600 nm. The nanosize of particles allows interaction with biomolecules within the cell and on the surface in such a way that could be designated to physiochemical properties of the cells [157]. Their potential application in drug delivery offered several advantages over conventional strategies. To utilize nanoparticles for drug delivery, it is important that these particles should be stable at nanoscale, biocompatible, and selectively directed to specific sites in the body after systemic administration. It could be achieved by conjugating the particle with ligand, which has a precise binding ability with respects at surface of targeted cells. In addition, nanoparticles could also bind specifically with therapeutic agents, hence increasing the concentration of therapeutic substances at the tendon injured site [157, 158].

For treatment of tendinopathy, nanoparticles can be used as nanometric delivery systems. For example, they are capable to enhance drug delivery through the skin via phonophoresis and iontophoresis techniques. Generally, both techniques are used to treat inflammatory conditions in tendon injuries. High-frequency ultrasound waves are used in phonophoresis to deliver drugs, while low-voltage current is used in case of iontophoresis. Dohenert et al. demonstrated that the improved delivery of diclofenac diethylammonium with phonophoresis and iontophoresis and gold nanoparticles (GNPs) as a drug carrier decreased the inflammatory response (reduction in TNF-*α* and IL-1*β* levels) in a tendinopathy animal model. The study concluded that GNPs could enhance the therapeutic effect of phonophoresis and iontophoresis by improving drug delivery and synergistic action of anti-inflammation [153, 159].

Moreover, the nanoparticles can be used as nonviral nanocarriers in gene therapy to deliver miRNAs *in vivo* and avoid peritendinous adhesion formation [160]. Zhou et al. showed that PEI-PLGA nanoparticles loaded in plasmid-inserted miRNA minimize the expression of TGF-*β*1. However, significant healing of injured tissue was not achieved, thus suggesting that the simultaneous delivery of a suitable combination of TGF-*β*1-miRNA and miRNAs of other growth factors is required. The strength of treated tendons was inferior as compared with the control group because of downregulation of cell proliferation, migration, adherence, and secretion of ECM-related to the inhibition of TGF-*β*1 [60, 160]. Mesoporous silica nanoparticles (MSN) with L-histidine can be also used for the treatment of tendinopathies. MSN significantly increased the efficiency of histidine functionalized nanoparticles in transfected cells as compared with imidazole or amino functionalized MSN [161].

Bioscaffolds can be coupled with nanoparticles to enhance regenerative properties of tendon healing tissue [21]. In these attempts, PLLA fibrous membranes, impregnated with dextran glassy nanoparticles loaded with *β*-FGF (dgNPs-FGF), were shown to stimulate cellular proliferation, differentiation, angiogenesis, migration, and ECM synthesis *in vitro and in vivo* of tendons (Achilles' tendon of Sprague-Dawley rat) [73]. It was concluded that dgNPs-FGF-loaded PLLA membrane can protect the bioactivity of *β*-FGF in a controlled manner to enhance the quality of healed tendon tissue.

## 12. Conclusion and Future Perspectives

Countless efforts have been made by scientists, but the treatment of tendon injuries still remains a challenge. Usually, repaired tissue has inferior structural and biomechanical properties and even long-term complications. An efficient strategy is urgently needed for tendon tissue engineering in view of sharp increase in tendinopathies in recent decades. This review has covered recent advances in the field of regenerative medicines, described scaffolds in terms of material science as new treatment options for tendon tissue engineering, and focused on identifying various aspects of biomaterials and biomechanics of tendon tissue repair. Our understanding regarding tendon embryonic development and repair mechanisms is still limited as well as the role of inflammation processes at critical times, the involvement of specific growth factors/immune modulators, and the regulation of genes during the natural healing processes. The lack of rational assessment tools is also a handicap to judge the properties of the repaired tissue; biomechanical parameters and patient's compliance are not reliable indicators for consolidation and advancement. Armed with more realistic biomechanical and biological assessment techniques, scientists will then be able to better evaluate the effects of regenerative medicines on the repaired tissue. The focal point of future studies is the discovery of an ideal combination of genes, proteins, and cells seeded on proper physical support, thus resulting in a scaffold that can mimic the native preinjured tendon architecture. Future investigations must be focused on the identification of nonimmunogenic, nontoxic, bioresorbable, and scalable biomaterials with ability to deliver growth factors and stimulate gene expression, cell proliferation, and differentiation. It is also our opinion that the synergistic combination of nanotechnologies with advanced 3D scaffold can provide a huge advancement in tendon regeneration research, thus speeding up the transfer of regenerative medicine efforts from bench to bedside.

## Figures and Tables

**Table 1:** Gene therapy for natural healing of the tendon.

Genes	Delivery method	Function	Reference
*Tnmd*	Transfection	Major constituents of collagen fiber. Positively regulated by Scx	[47]
*Scx*	Polymersome scaffolding material	Major marker of tendon & ligament tissues. Encode protein which expressed during embryonic development	[13]
*Fmod*	Carried by fibrin gel	Positively regulate expression of TGF-*β*. Encode protein which play role in ECM deposition	[47]
*COL-I*	Transfection	Major constituents of ECM	[162]
*COL-III*	Transfection	Major constituents of ECM	[162]
*COL-V*	Transfection	Constituent of ECM and play role in fiber strength	[163]
*LAMA-4*	Fibrin-heparin-based delivery	Encodes laminin alpha-4, noncollagenous constituent of ECM	[164]
*ELN*	Liposomal nanoparticles	Encodes elastin protein, major structural constituent of ECM	[164]
*FBN-II*	Carried by fibrin gel	Encodes fibrillin-II protein, major structural constituent of ECM	[164]
*Comp*	Nanoliposomes	Belongs to the ECM protein. Structural role	[165]
*Decorin*	PLGA-NP	Constituent of ECM structural protein, positively regulated by TGF-*β*, and enhance strength and organization of collagen fibers	[160]

Abbreviations: Tnmd: tenomodulin; Scx: scleraxis; Fmod: fibromodulin; COL-I: collagen-I; LAMA-4: laminin subunit alpha-4; ELN: elastin gene; FBN-II: fibrillin-II; Comp: cartilage oligomeric matric protein.

**Table 2:** Tendogenic growth factors.

Growth factors	Study type	ECM production	Tendon model	Results	Reference
TGF-*β*	*In vitro*	Increased expression of collagen-I & III	Equine embryo	TGF-*β* promote differentiation and proliferation	[166]
PRP	*In vitro*	Not studied	Equine superficial digital flexor tendon	Increased cellularity, GAG contents, tensile strength and matrix strength	[138]
VGF-11	*In vitro*		Rat Achilles' tendon	Promote proliferation, increased mechanical and tensile strength	[167]
IGF-1	*In vitro*	Enhanced orientation of collagen fibers	Rabbit patellar tendon	Promote formation of fibrous tissue with increase crosslinking and orientation	[119]
BMP-12	*In vitro*	Increased expression of collagen-I	Human ADSC	Regulate expression of Scx and RUNX and promote tenogenesis	[168]
FGF-2	*In vitro*	Not studied	Rat TSPCs	Promote cell proliferation up till week 6 and regulate expression level of Tnmd & Scx	[151]
bFGF	*In vitro*	Increased expression of collagen-III	Rat patellar tendon	Increased cell differentiation and collagen content	[169]
GDF-5	*In vitro*	No significant difference found as compared with control	Human BMSCs	GDF-5 promote differentiation in BMSCs but significant effect on proliferation was not observed, induce tenogenic differentiation without cell doubling	[170]
GDF-6	*In vitro* & *in vivo*	Increased deposition and organization of collagen	Rabbit BMSc	Promote cell differentiation, and regulate expression of Tnmd & Scx	[171]
GDF-7	*In vitro*	Not studied	Equine BMSc	Regulate expression of decorin & Tnmd and cell differentiation	[172]

Abbreviations: TGF-*β*: transforming growth factor beta; PRP: platelet-rich plasma; VGF-11: nerve growth factor inducible; IGF-1: insulin-like growth factor-1; BMP-12: bone morphogenic protein-12; FGF-2: fibroblast growth factor-2; bFGF: basic fibroblast growth factor; GDF-5: growth differentiation factor-5.

**Table 3:** Recent cell-based approach for tendon regeneration.

Cell line	Study type	Tendon model	Results	Reference
Tenocytes	Rabbit Achilles tendon	*In vivo*	Increased ECM production and enhanced orientation of collagen fibers	[82]
ADSC	Rabbit Achilles tendon	*In vitro/in vivo*	Production of tendon fibers with better (60%) tensile strength	[128]
Dermal fibroblast	Human patella refractory tendinopathy	*Clinical trial*	Increased tendon thickness, reduction in pain and enhanced mechanical strength	[131]
BM-MSC	Equine tendon model	*In vitro/in vivo*	Improved histological parameters, increased orientation of collagen fibers and decrease in cellularity	[141]
Muscle-derived stem cells	Muscle muscularis fascia of dorsum	*In vivo*	Enhanced stiffness, tensile strength and improved cross linking of collagen fibers	[90]
Periosteal progenitor cells	Rabbit infraspinatus tendon model	*In vivo*	Increased content of col-I & COL-II and enhanced ECM production	[128]
Tendon stem cells	Rat patella tendon model	*In vitro/in vivo*	Increased collagen fiber thickness, ECM deposition and production of col-I, II and Tnmd	[93]

Abbreviations: ADSC: adipose-derived stem cells; BM-MSC: bone marrow-derived mesenchymal stem cells.

## Data Availability

Because this is a review paper, no research data is included in the manuscript.
